# Maternal genomic variability of the wild boar (*Sus scrofa*) reveals the uniqueness of East‐Caucasian and Central Italian populations

**DOI:** 10.1002/ece3.5415

**Published:** 2019-07-27

**Authors:** Saber Khederzadeh, Szilvia Kusza, Cui‐Ping Huang, Nickolay Markov, Massimo Scandura, Elmar Babaev, Nikica Šprem, Ivan V. Seryodkin, Ladislav Paule, Ali Esmailizadeh, Hai‐Bing Xie, Ya‐Ping Zhang

**Affiliations:** ^1^ State Key Laboratory of Genetic Resources and Evolution, Kunming Institute of Zoology Chinese Academy of Sciences Kunming China; ^2^ Kunming College of Life Science University of Chinese Academy of Sciences Kunming China; ^3^ University of Chinese Academy of Sciences Beijing China; ^4^ Animal Genetics Laboratory, Faculty of Agricultural and Food Sciences and Environmental Management University of Debrecen Debrecen Hungary; ^5^ Department of Game Animals' Ecology, Institute of Plant and Animal Ecology Ural Branch of Russian Academy of Sciences Yekaterinburg Russia; ^6^ Department of Veterinary Medicine University of Sassari Sassari Italy; ^7^ Caspian Institute of Biological Resources Makhachkala Russia; ^8^ Department of Fisheries, Beekeeping, Game Management and Special Zoology, Faculty of Agriculture University of Zagreb Zagreb Croatia; ^9^ Pacific Geographical Institute FEB RAS Vladivostok Russia; ^10^ Far Eastern Federal University Vladivostok Russia; ^11^ Faculty of Forestry Technical University in Zvolen Zvolen Slovakia; ^12^ Department of Animal Science, Faculty of Agriculture Shahid Bahonar University of Kerman Kerman Iran

**Keywords:** Caucasus, migration modeling, phylogeny, *Sus scrofa*, whole mtDNA

## Abstract

The phylogeography of the European wild boar was mainly determined by postglacial recolonization patterns from Mediterranean refugia after the last ice age. Here we present the first analysis of SNP polymorphism within the complete mtDNA genome of West Russian (*n* = 8), European (*n* = 64), and North African (*n* = 5) wild boar. Our analyses provided evidence of unique lineages in the East‐Caucasian (Dagestan) region and in Central Italy. A phylogenetic analysis revealed that these lineages are basal to the other European mtDNA sequences. We also show close connection between the Western Siberian and Eastern European populations. Also, the North African samples were clustered with the Iberian population. Phylogenetic trees and migration modeling revealed a high proximity of Dagestan sequences to those of Central Italy and suggested possible gene flow between Western Asia and Southern Europe which was not directly related to Northern and Central European lineages. Our results support the presence of old maternal lineages in two Southern glacial refugia (i.e., Caucasus and the Italian peninsula), as a legacy of an ancient wave of colonization of Southern Europe from an Eastern origin.

## 1 INTRODUCTION

Wild boar (*Sus scrofa*) is widely distributed all over the world, and their population is almost four million, hence, considered the second most abundant ungulate species in Europe and a pest in several regions (Apollonio, Andersen, & Putman, 2010; Bobek, 2018). However, some authors have attributed their range expansion to climate change (mild winters with no or low snow cover, triggering the necessity for higher food intake; Jędrzejewska, Jędrzejewski, Bunevich, Miłkowski, & Krasiński, 1997; Root et al., 2003; Vetter, Ruf, Bieber, & Arnold, 2015). Others argue that this population increase has been caused by intensive crop production, changes in agricultural practices, reduced number of predator species, declined hunting pressure, human activities such as supplementary feeding, intentional releases for hunting purposes, and hybridization with domestic pigs (Apollonio, Scandura, & Šprem, 2014; Baskin & Danell, 2003; Bieber & Ruf, 2005; Geisser & Reyer, 2004, 2006; Iacolina, Corlatti, Buzan, Safner, & Šprem, 2019; Jędrzejewska et al., 1997; Leaper, Massei, Gorman, & Aspinall, 1999; Massei & Genov, 2004; Massei et al., 2015; Sáez‐Royuela & Tellería, 1986).

Genetic studies can be used to discover more diversity patterns as well as in explaining the changes observed in the species' geographical range. Throughout its history, the wild boar has been strongly influenced by human practices such as hunting, pig domestication, and animal translocation (Larson et al., 2005; Scandura, Iacolina, & Apollonio, 2011). Thus, analysis of the changes in the geographical range of *S. scrofa* could aid our understanding of not only the global patterns of evolution and ecosystem change (Anijalg et al., 2018). Previous studies revealed the complex genetic structure of wild boar populations in Eurasia, including multiple domestication events and gene flow between wild boar and domestic pig breeds (Iacolina et al., 2016; Larson et al., 2005; Ramíres et al., 2009; Ribani et al., 2019; Šprem et al., 2014).

Since the most of these studies had been focused on Europe or Eastern Asia (Kusza et al., 2014; Larson et al., 2005; Scandura et al., 2011; Veličković et al., 2015; Vilaça et al., 2014), the specific status and phylogenetic position of West Asian pigs have only recently been reported. A recent investigation by Khalilzadeh et al. (2016) revealed the presence of Middle Eastern, European, and East Asian haplotypes in Iranian wild boar and proposed that there had been contact between the European and East Asian wild boar populations. Thus, analysis of samples from a large number of geographical localities, across different regions, could provide a more comprehensive description of the evolutionary history of wild boar.

A factor that could affect the quality of analysis is the type of genetic marker used in any study. Most genetic studies on the diversity or phylogenetics of wild boar were based on partial D‐loop sequences of mitochondrial DNA (mtDNA; less than 7% of the whole mtDNA genome), or cytochrome b sequences (Fang & Andersson, 2006; Kusza et al., 2014; Larson et al., 2005; Ramíres et al., 2009; Scandura et al., 2008; Veličković et al., 2015, 2016; Vilaça et al., 2014), which occupies less than 7% of the whole mtDNA genome, sometimes in combination with another region (e.g., cytochrome b). To the best of our knowledge, there are only a few previous examples of using a complete mtDNA genome to assess the phylogeographical relationships of *S. scrofa*. Ni et al. (2018) addressed their global phylogeography, based on sequences data from domestic pigs (72%), and a comparatively low number of wild boar samples. The authors showed patterns similar to those obtained by use of partial mtDNA genome sequences, thus demonstrating a clear separation of European and Asian clades. Chen et al. (2018) addressed the relationships between North Asian and South Asian wild boars, based on the effect population size (Ne) and climate on the non‐synonymous/synonymous mutation ratio (Ka/Ks). Thus, there is a gap in genetic studies of wild boar, due to the lack of complete mtDNA on the Western part of the species' geographical range.

In this study, we present an analysis of SNPs variability in the whole mtDNA genome of wild boar from North Africa and continental Eurasia (various regions from Western Europe to Western Siberia). Firstly, we aimed to determine the phylogenetic position of animals from Eastern Caucasus—a region not previously included in any phylogeographical study. Secondly, we addressed the question of how a phylogenetic tree of wild boar, based on SNPs of whole mtDNA genome data, would differ from a phylogenetic tree based on shorter fragments (D‐loop and cytochrome b markers).

## 2 MATERIALS AND METHODS

### 2.1 Ethics statement

We declare that we have no financial or personal relationships with other people or organizations that can inappropriately influence our work, and that we have no professional or other personal interest of any nature or kind in any product, service, and/or company that could be construed as influencing our research.

No wild boars were culled solely for the purpose of the present study. Tissue samples from each different country were obtained from collaborators and hunters. All samples were collected in compliance with each county's national regulations on wild boar management plans.

### 2.2 Material

A total of 77 samples (fresh muscle *n* = 72, blood *n* = 5) were collected in 2014–2017 in Europe (Germany *n* = 7, Poland *n* = 7, Italy *n* = 6, Bulgaria *n* = 5, Hungary *n* = 5, Ukraine *n* = 5, Netherlands *n* = 4, Croatia *n* = 4, Estonia *n* = 4, Romania *n* = 4, Slovakia *n* = 4, Czech Republic *n* = 4, Spain *n* = 3, France *n* = 1 and Switzerland *n* = 1), Asian (Western Siberia *n* = 4), and East‐Caucasus (Dagestan *n* = 4) parts of Russia and North Africa (Morocco *n* = 4 and Tunisia *n* = 1).

Tissue samples were stored in plastic tubes (5–30 ml) filled with 96% ethanol. Blood samples were kept frozen in EDTA tubes at −20°C until further analysis. Laboratory experiments were performed at the laboratory of Molecular Evolution and Genome Diversity of State Key Laboratory of Genetic Resources and Evolution, Kunming Institute of Zoology, Chinese Academy of Sciences. Available whole mtDNA sequence data of for five suids warthog (*Phacochoerus africanus*), Javan warty pig (*Sus verrucosus*), Visayan warty pig (*Sus cebifrons*), Celebes warty pig (*Sus celebensis*), and Bornean bearded pig (*Sus barbatus*) were downloaded, to be used as outgroups, from the NCBI database (accession numbers: ERR173209, ERR173210, ERR173177, ERR173211, ERR173203). These five genomes were combined with the 77 mtDNA genomes obtained in this study for further statistical analyses.

### 2.3 Laboratory methods

In this research, some samples were extracted with QIAamp DNA Blood Spin kits (Qiagen), and most of which were extracted using the phenol–chloroform method (Sambrook, Fritsch, & Maniatis, 1989). The quality and quantity of the extracted DNA were checked using a NanoDropTM 8000 spectrophotometer (NanoDrop Technologies Inc.). All experiments were performed according to the ethical regulations of the Chinese Academy of Sciences (Approval ID: SYDW‐2015012).

### 2.4 Sequence analysis

Library preparation for the Illumina sequencing platform required fragmentation of our DNA (1–3 μg of genomic DNA), followed by repair of 3′ and 5′ ends to form blunt‐ended, phosphorylated molecules, and the addition of a non‐templated dA‐tail before ligation to an adaptor. DNA libraries were prepared according to the standard Illumina library preparation protocol, with a short insert size range of 300–500 bp, and then sequenced on an Illumina HiSeq 2000 platform with the 150 bp paired‐end sequencing kits. Pair‐end reads (150 bp) were sequenced to about 10× sequencing depth and ≥99% coverage for each individual.

Quality control procedure was used to remove reads with low sequencing quality. Particularly, reads were trimmed for minimum Phred quality >20 over three consecutive base pairs and discarded if shorter than 45 bp. Clean reads were trimmed from raw reads that were preprocessed to remove index adaptors and low‐quality reads. Quality control for removing the low‐quality reads was done based on the following criteria: up to 10% of the read bases include “N” content of each sequenced reads, up to 50% of the read bases include low‐quality (*Q* <= 5) base content in any sequenced reads, and finally, by removing duplicate reads, using Picard tools v.2.12.1.

After quality trimming, clean reads of each sample were aligned against the mitochondrial genome from the domestic pig reference genome (*S. scrofa* 10.2, which was downloaded from Ensembl genome browser) using Burrows‐Wheeler Aligner (BWA) software (Li & Durbin, 2010). After extracting whole mitochondrial genome from our raw data by VCFtools 1.13 (Danecek et al., 2011), variants were called using Genome Analysis Toolkit (GATK) (Nekrutenko & Taylor, 2012). To avoid potential bias between our extracted data and the publicly available data, we called SNPs by comparing the mitogenome sequence of each individual to the mitochondrial reference genome and then merged the called SNPs to form a common set of SNP data for the 82 individuals (including 77 samples of wild boar and five sequences for outgroups). Then, several filtering steps were applied before using candidate SNPs for further analyses to minimize the number of false positive calls. Then, we removed all of the missing sites and also SNPs with minor allele frequency of 0.01, leaving 933 SNPs for next step. Afterward, the Integrative Genomics Viewer 2.5 (IGV) (Robinson, Thorvaldsdóttir, Wenger, Zehir, & Mesirov, 2017) was used as a high‐performance visualization tool for interactive exploration of large, integrated genomic datasets and to confirm reliability of high‐quality polymorphic sites (269 SNPs) with no any linkage makers based on reference genome for further analyses.

### 2.5 Statistical analyses

Single nucleotide polymorphism sequences from the whole mtDNA were aligned using the Clustal W software (Thompson, Gibson, Plewniak, Jeanmougin, & Higgins, 1997) implemented in MEGA v.6 software (Tamura, Stecher, Peterson, Filipski, & Kumar, 2013). Levels of molecular diversity such as haplotype number (*h*), haplotype diversity (*H*_d_), nucleotide diversity (*π*), Fu's *F*_S_, and Tajima's *D* were independently computed with DnaSP v.5.10 (Librado & Rozas, 2009) for 77 samples of *S. scrofa*.

The Bayesian phylogenetic tree was constructed using 78 sequences (adding 1 sequence of warthog [*P. africanus*] as an outgroup) using MrBayes 3.2.2 software (Ronquist & Huelsenbeck, 2003). The evolutionary parameters were given by jModeltest2.1 (Posada, 2008). Two independent Monte Carlo Markov Chains (MCMC) were run for 50,000,000 generations, with sampling every 100 generations and discarding the first 10% as burn‐in. The resulting trees were visualized using FigTree 1.4.2 (http://tree.bio.ed.ac.uk/software/figtree/).

Further phylogenetic analyses were performed using neighbor‐joining method, based on the genetic distance matrix in mega v.6 software (Tamura et al., 2013). Maximum likelihood tree (ML) was constructed with 1,000 bootstrap replicates using treemix v.1.1 when the migrations were prohibited. For both phylogenetic trees (NJ & ML), sequences of five suids were used as outgroups (*n* = 82).

For a large number of haplotypes, the complexity of a network can be solved with a median‐joining (MJ) network algorithm. So, based on all haplotypes of the complete mtDNA, a MJ network was constructed using network, version 5.0.0.3 (Bandelt, Forster, & Röhl, 1999). We also performed a principal component analysis (PCA) with population‐scale SNPs, as implemented in the smartPCA program in the eigensoft package v.6.1.4 (Galinsky et al., 2016; Patterson, Price, & Reich, 2006; Price et al., 2006).

treemix v.1.1 (Pickrell & Pritchard, 2012) was used to model demographic scenarios in the form of a bifurcating tree based on the maternal genome, allowing for inferring migration events between individuals and populations to provide insights into hidden demographic events of the past and testing for the effect and presence of gene flow between divergent populations. treemix software first builds a maximum likelihood tree of sampled populations by approximation of genetic drift. Subsequently, it identifies populations whose genetic covariance is underestimated by the model. Three migration events were selected on the basis of a different number of bootstraps to improve fitness of the model. To compensate for linkage disequilibrium, we applied strict filters to exclude all positions with genetic linkage. As the use of our dataset would require input file to be in to Ped/Map formats, we avoided any interference of linked markers by applying ‐‐indep‐pairwise parameter in PLINK v.107 (Purcell et al., 2007) for windowed pruning of the data to obtain a pruned file which we then used to get second Ped/Map files, the final input for subsequent analyses. Therefore, our analysis was based on non‐link markers. Together, the nature of our dataset, Biallelic SNP VCF of the whole mtDNA, and data filtering steps made it possible for us to reliably use treemix.

## 3 RESULTS

### 3.1 Mitogenome variability of wild boars

**Figure 1:**
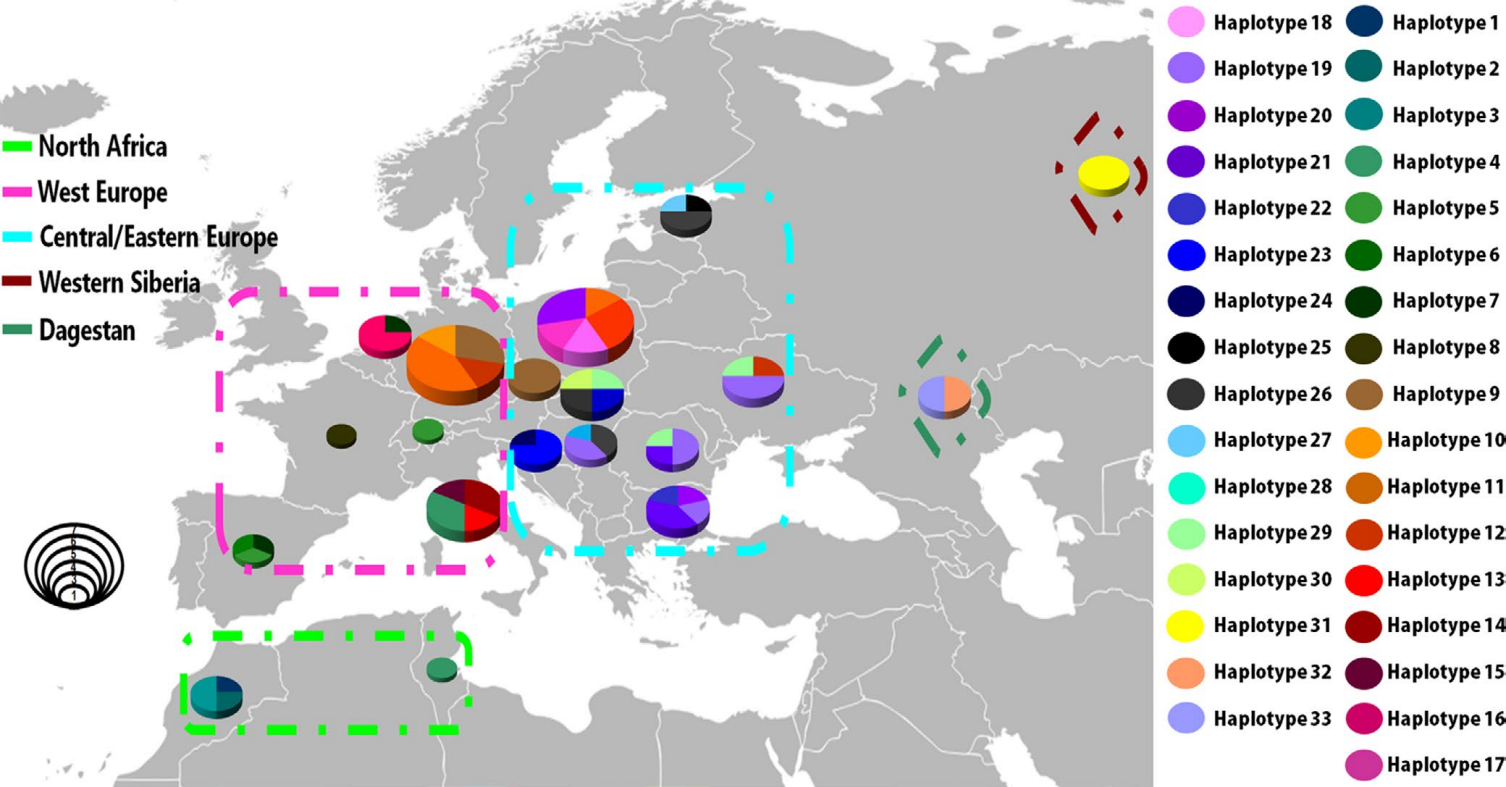
Geographical distribution of the sequenced haplotypes. Areas of circles on the map are proportional to the number of sampled individuals. Dotted lines represent geographical regions

A total of 77 complete wild boar mtDNA genomes from Europe (*n* = 64), North Africa (*n* = 5), and Russia (*n* = 8) were sequenced for this study. After the quality control steps, 269 polymorphic sites were retrieved for the analyses. A total of 33 haplotypes were detected in our dataset, of which 27, 4 and 3 were found in Europe, North Africa and Russia, respectively (Figure 1, Table 1). Seventy‐nine percent of haplotypes were found in just one region, namely Europe. The most widely distributed haplotype was Hap12, which was found in Western, Central, and Eastern Europe. The proportion of unique haplotypes was 0.54. The haplotypes shared by the highest number of individuals were Hap 19, found in 10 animals in Central and Eastern Europe and Hap9 found in 7 animals in Western and Central Europe. The haplotype diversity was generally high (over 0.9) in North Africa and Europe, while for Western Siberia and Eastern Caucasus it was relatively low (Table 1). The highest values of both haplotype and nucleotide diversity were found in Western Europe. We did not detect a significant departure from the neutrality expectation, as calculated by both Fu's *F*_S_ and Tajima's *D* (Table 1).

**Table 1:** Basic parameters for genetic (mtDNA) variability within wild boar populations

Populations	Number of samples	Number of different haplotypes	Detected haplotypes (Hap)	Haplotype diversity ± *SD*	Variance in haplotype diversity	Nucleotide diversity ± *SD*	Tajima's *D*	Fu's *F*s	Number of polymorphic sites
*North Africa*	5	4	Hap1/Hap2/Hap3/Hap4	0.900 ± 0.161	0.025	0.002 ± 0.000	−0.894^ns^	0.212^ns^	10
*West Europe*	22	13	Hap4‐16	0.944 ± 0.026	0	0.015 ± 0.004	−0.843^ns^	3.987^ns^	137
*Central and Eastern Europe*	42	17	Hap9/Hap11/Hap12/Hap17‐30	0.912 ± 0.026	0	0.004 ± 0.000	−1.491^ns^	−0.931^ns^	58
*Western Siberia (Russia)*	4	1	Hap31	N/a	N/a	N/a	0	N/a	N/a
*Eastern Caucasus (Dagestan, Russia)*	4	2	Hap32/Hap33	0.667 ± 0.204	0.041	0.001 ± 0.000	2.080^ns^	2.719^ns^	4

Note

The details of five main populations are in italics font.

### 3.2 Phylogenetic analysis

**Figure 2:**
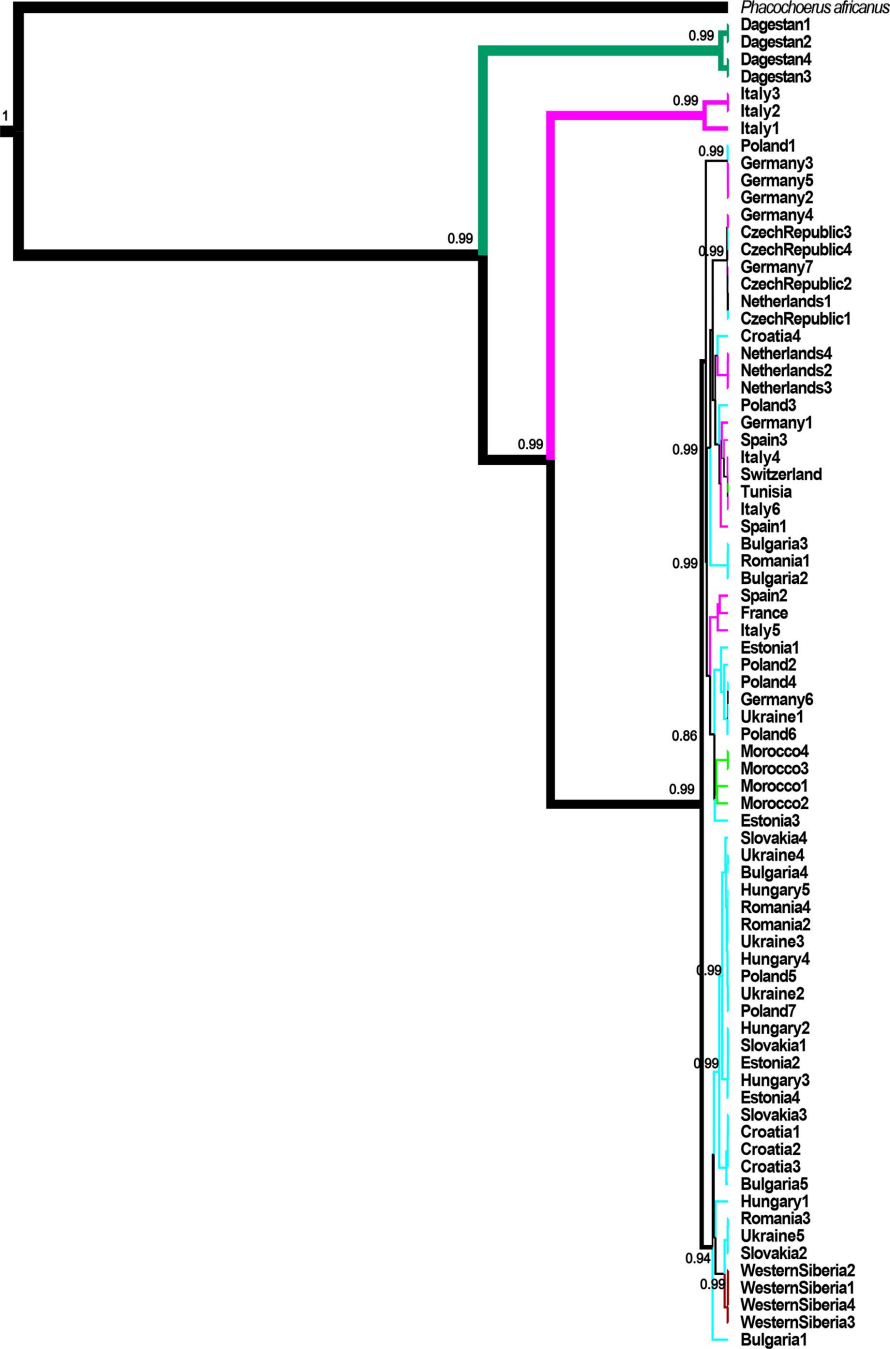
Phylogenetic relationships of the studies samples basing on the Bayesian phylogenetic tree. Posterior probabilities ≥0.8 are given. The numbers on the branches are posterior probabilities from the Bayesian inference. Different colored lines represent the clusters of populations geographically close to each other (green indicates North Africa; pink indicates West Europe; light blue indicates Central Europe and Eastern Europe; dark red indicates Western Siberia; dark green indicates Dagestan)

Phylogenetic (NJ, Maximum likelihood, Bayesian) trees and median‐joining networks (MJ) were constructed using SNPs of sequenced mtDNA genomes from the present study and mitochondrial genomes of five *Suidae* species across the World. Both NJ, ML (Appendix 1) and Bayesian (Figure 2) trees showed similar topologies. In the Bayesian tree, the samples from Dagestan and some samples from Central Italy (Italy 1‐3, Hap13,14) were rooted directly in the basal branch and clearly separated from all other populations. All the other samples formed a single cluster where all haplotypes originated from the same root. West Siberian samples showed a phylogenetic position close to Eastern European (Ukrainian, Romanian) populations, while North African samples were close to the Spanish population.

**Figure 3:**
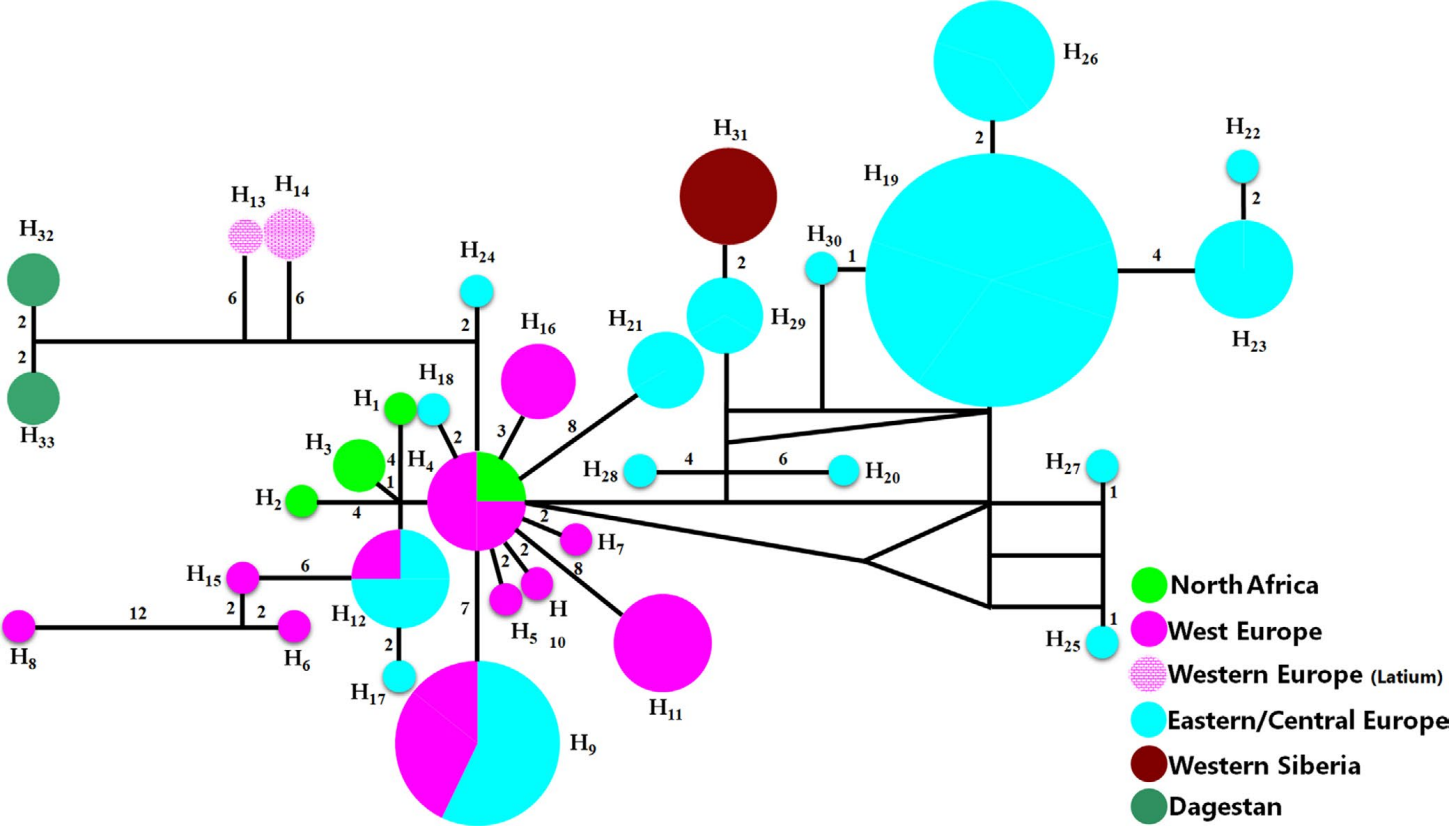
Median‐joining networks based on SNPs of complete mtDNA. The network was constructed with 77 wild boar sequences. Mutations are represented by the numbers in which they occurred. Geographical locations of samples are represented by circles with color, with sizes that are in proportion to the number of sampled individuals

The MJ network (Figure 3) also shows the distal position of the East‐Caucasian and Central Italian samples to other haplotypes. This analysis also revealed the geographical structure within the group of European and North African samples. They form two star‐shaped structures. One consists of haplotypes found in Western Europe and Northern Africa, while the other includes haplotypes from Central and Eastern Europe and from Western Siberia. The “central” haplotypes in the Western European and the Eastern European clusters are Haplotype 4 (found in Italy and Switzerland) and Haplotype 19 (found in Romania, Poland, Hungary, and Ukraine).

### 3.3 Principal component analysis and gene flow

**Figure 4:**
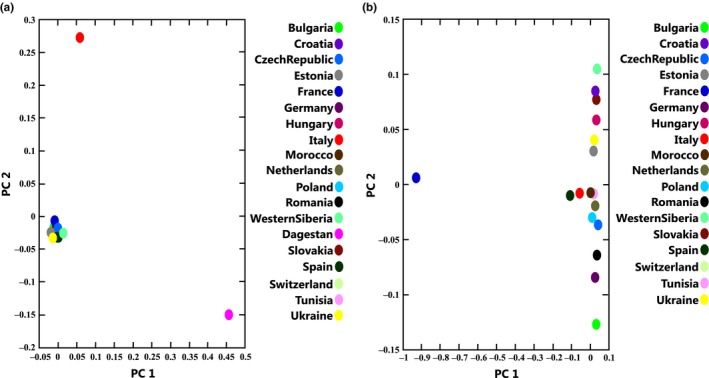
PCA for different subpopulations. (a) PCA result for 77 samples belonging to 19 subpopulations. (b) PCA result for 70 samples belonging to 18 subpopulations (4 samples from Dagestan and 3 from Italy were excluded). PC1, principal component 1; PC2, principal component 2

In the principal component analysis, the variation along the first principal component (PC1) explained 51.62% of the variance and separated the East‐Caucasian samples from all the other geographical regions. The variation along the second principal component (PC2), explained 22.93% of the variance and showed the specificity of the Italian (Latium) population in comparison with all the other samples (Figure 4a). If the PCA was run excluding all Dagestan and samples from Latium, Italy, it did not demonstrate any clear geographical structure (Figure 4b), except the distal position of French samples to the position of other European samples.

**Figure 5:**
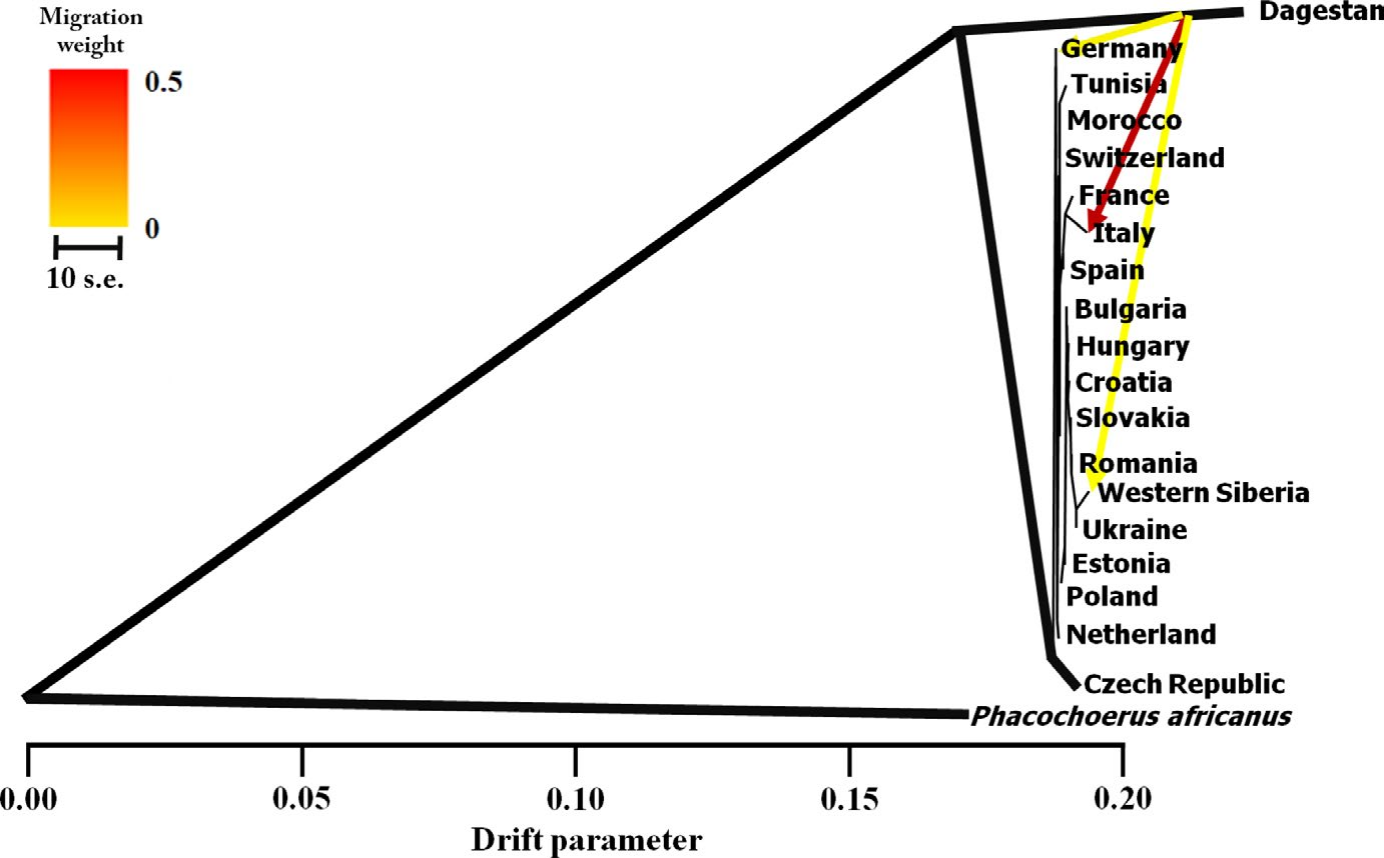
Maximum likelihood tree depicting the genetic relationships between 19 *Sus scrofa* populations. Results were based on treemix for all populations, allowing for three migration events. The weight of the migration component follows the key on the left. The scale bar shows ten times the average standard error of the entries in the sample covariance matrix

We also studied population splits and gene flow between wild boar populations. Using treemix, we firstly constructed a phylogenetic tree where no migration event was allowed. Here, outgroup and Dagestan were separated from other populations. Then, up to ten migration events were added to the tree. This phylogenetic tree showed that the Dagestan population was clearly separated from other populations (Figure 5). treemix runs showed a high genetic affinity between Dagestan samples and the Italian samples (according to the model 79.14% of alleles in Central Italian clade were descended from Dagestan clade) and evidence of possible very weak gene flow not only between Dagestan and Western Siberia, but also between Dagestan and Germany (3.1% and 3%, respectively).

## 4 DISCUSSION

Most published studies of the genetic diversity and phylogenetic relationships among wild boar mtDNA haplogroup lineages have been performed using the D‐loop or the cytochrome b regions of the mtDNA genome (Alexander, Novembre, & Lange, 2009; Kusza et al., 2014; Larson et al., 2005; Scandura et al., 2008; Veličković et al., 2015). Wild boars have an intermediate level of mtDNA D‐loop variability (Djan et al., 2013), and little information is available about their complete mtDNA genome diversity (Chen et al., 2018; Kijas & Andersson, 2001; Ni et al., 2018; Tan et al., 2018). However, the analysis of whole mtDNA genome could lead to new and important inferences about the origin and diversification of *S. scrofa* populations. Here, we studied the SNPs of complete mtDNA genome and variability of wild boars from West Siberia, Eastern Caucasus, Europe, and North Africa to provide new data on the species' genetic diversity and phylogenetic patterns. The results of our study revealed the peculiar haplotype composition of the wild boar inhabiting two specific areas: East‐Caucasus (Dagestan) and Central Italy. For the first time, we have detected unique mtDNA haplotypes from Dagestan that could probably appear early in the wild boar phylogeny. The genetic uniqueness of Italian wild boars has already been shown in previous studies (Larson et al., 2005; Scandura et al., 2008), indicating that the Italian peninsula has served as a Southern refugia during the Pleistocene glaciations, whereas the Alps may have acted as a barrier to gene flow (Scandura et al., 2008; Veličković et al., 2015; Vilaça et al., 2014). Most interesting was the similarity of Central Italian and Caucasian haplotypes (as shown by treemix analysis, Figure 5). A similar trend involving the proximity of Caucasian and Mediterranean haplotypes has also been reported for common hamster (*Cricetus cricetus*), by Feoktistova et al. (2017). This could explain the presence of two genetic lineages in the territory of modern Italy, as shown by a number of previous studies (Larson et al., 2005; Scandura et al., 2008). High genetic diversity in Southern Europe, particularly in Italy, has been explained by the preservation of lineages during glaciation in several Pleistocene refugia. Another genetically distinct lineage (commonly known as E1 and found over the whole of Europe) could have arrived to Italy from Northern Europe during the contraction of range during the period of glaciation.

The hypothesis of migration of West Asian haplotypes to Europe and particularly to Italy is supported by the results of other researchers. Particularly, Alexander et al. (2009) have reported Near Eastern haplotype on the island Samos, while Veličković et al. (2015) have also reported Asian haplotype from the South of Balkan Peninsula. According to Maselli et al. (2016), the proportion of “Asian” haplotypes in Southern Italy and Sardinia was about 9%. Manunza et al. (2013) based on the analysis of autosomal SNPs also suggested the scenario of migration from Trans‐Caucasus region and Western Asia to Western Europe.

The lack of distinct clades (similar to E1 and E2) on the Balkan Peninsula (Alexander et al., 2009; Veličković et al., 2015) could be treated as contradicting the hypothesis of wild boar expansion from West Asia to Southern Europe through Asia Minor. Thus, the South Balkans haplogroup may represent a remnant of the pre‐LGM gene pool of the Balkans, in the same way as the European E2 haplogroup is a remnant of pre‐LGM diversity in Italy (Veličković et al., 2015). However, it is possible that on the Balkans the haplotypes that arrived from West Asia could be replaced by haplotypes described as the E1 group. In contrast to the Balkans, Italy is separated from Northern Europe by the Alps, which extend in an east–west direction. This could have reduced the expansion of northern wild boars to the Apennine peninsula and have helped preservation of lineages, which hypothetically could arrive from the South.

The genetic proximity of Caucasian and South European samples could also be related to direct translocations of pigs from West Asia to Southern Europe. Maselli et al. (2016) suggested that the Near Eastern haplotypes found in the ancient pigs of Italy could have descended from early domesticated pigs that arrived into Europe during the Neolithization of the continent or from a legacy of previous contacts with some northern populations. However, Vai et al. (2015) suggested reconsidering this hypothesis. Based on the analysis of ancient samples, they showed that the E2 clade was present in Italy before the NE haplotypes arrived to Southern Europe, and the NE haplotypes in turn were dated to the pre‐Neolithic period, thus prior to domestication. Further studies of central Asian wild boar are needed to clarify this subject. It will also be important to study the genetic relationships between Caucasian and East Asian wild boars.

Weak genetic drift from Caucasus to Western Europe (Germany) indicates that Dagestan clade did not contribute significantly to North and West European genetic lineages. This result supports the hypothesis of expansion of West Asian haplotypes to Southern Europe through Asia Minor, but not through Northern Europe.

The genetic proximity of West Siberian wild boars to European groups can be accounted by the fact that West Siberian population was established as a result of intentional releases of animals presumably from the European part of Russia, Eastern Europe (Belarus), and the Northern Caucasus (Markov & Bolshakov, 1996). Our results indicate that European lineages are more common in newly established populations of Western Siberia. These results are consistent with those obtained by Zinovieva (2013) using polymorphism of genes associated with quantitative trait loci. They showed that West Siberian animals grouped with European and Caucasian wild boar rather than with populations from the Trans‐Baikal region and the Far East part of Russia.

The topology of the Bayesian tree based on whole mtDNA genomes did not differ from trees created using the partial sequences on control region and cytochrome b, presented in previously published articles (Fang & Andersson, 2006; Kusza et al., 2014; Larson et al., 2005; Veličković et al., 2015). As in previous studies (e.g., Fang & Andersson, 2006; Larson et al., 2005; Soria‐Boix, Donat‐Torres, & Urios, 2017), this showed a lack of genetically specific groups within continental Western, Central, Eastern Europe, and Northern Africa (Morocco and Tunisia) and the specificity of the Italian population. On the other hand, the MJ network based on the whole mtDNA sequences suggests differences in haplotype composition between Western Europe and Central‐Eastern Europe. The division of samples on the MJ network is however in general agreement with clusters E1‐A and E‐1C described in Scandura et al. (2011). Clearly, there was no isolation between Western and Central‐Eastern Europe wild board populations, but the proportion of different haplotypes in these two regions and star‐shaped MJ networks raises the question of possible differences in origin of wild boars from different parts of the European continent.

We conclude that inclusion of the data from a previously unsampled region (Eastern Caucasus), using the complete mitochondrial genome as a basis for phylogeographical analysis and modeling animals' migrations, allowed us to confirm diversification of populations based on partial sequences of mitochondrial genome and hypothesize about possible ways of expansion in the western part of the *S. scrofa* geographical range. Further analysis of data collected over a larger area, and inclusion of ancient samples is needed to check these findings.

## CONFLICTS OF INTEREST

The authors declare no conflict of interests.

## AUTHOR CONTRIBUTIONS

The study was designed by YPZ, AE, SK, NM. SK, and CPH performed the molecular analysis. SK and Sz.K performed the population genetic analyses. SK, NM, MS, EB, NS, IVS, LP, AE, and HBX provided the data. NM, Sz.K, and SK wrote the paper and all authors read, corrected and approved the manuscript.

## DATA AVAILABILITY STATEMENT

Our novel data has an accession number of PRJEB30825 and is available in the European Bioinformatics Institute (EMBL‐EBI).

## Figures and Tables

**Figure A1:**
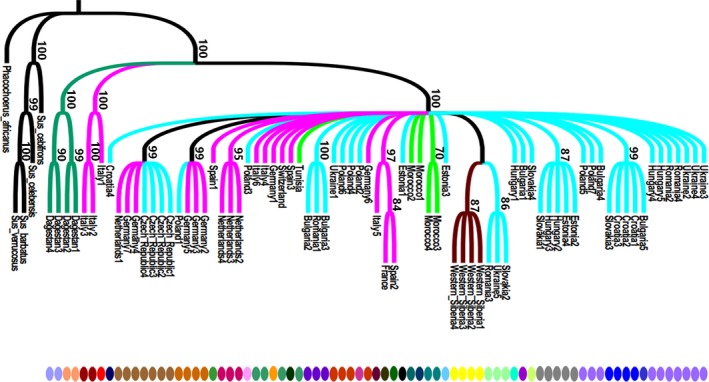
Neighbor‐joining tree (Tamura‐Nei model, 1,000 bootstrap replicates). Different colored lines represent the clusters of populations geographically close to each other (green indicates North Africa; pink indicates West Europe; yellow indicates Central Europe; light blue indicates Eastern Europe; orange indicates Estonia; purple indicates West Russia). Different colored circles represent each of 33 haplotypes

**Figure A2:**
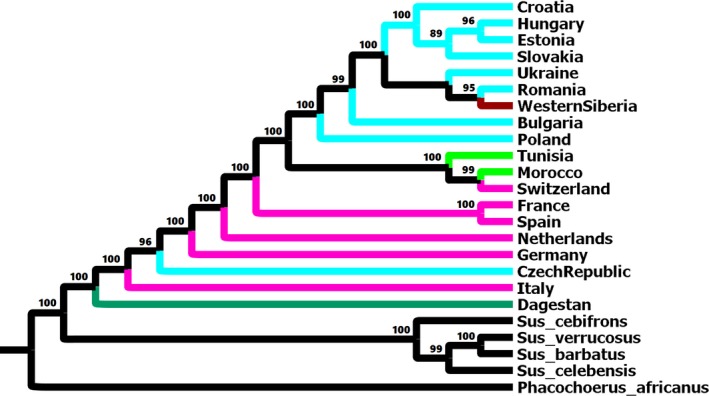
Maximum Likelihood tree, depicting the relationship between the SNPs of whole mtDNA genome sequences used in this study (1,000 bootstrap replicates). The colors of the branches represent the clusters of subpopulations (green indicates North Africa; pink indicates Western Europe; yellow indicates Central Europe; light blue indicates Eastern Europe; orange indicates Estonia; purple indicates Western Russia)
